# Standardized Welfare Terms for the Zebrafish Community

**DOI:** 10.1089/zeb.2016.1248

**Published:** 2016-07-01

**Authors:** Nicola Goodwin, Natasha A. Karp, Samuel Blackledge, Bradley Clark, Rosemary Keeble, Ceri Kovacs, Katrina N. Murray, Michael Price, Peter Thompson, James Bussell

**Affiliations:** ^1^ Research Support Facility, Wellcome Trust Sanger Institute, Cambridge, United Kingdom.; ^2^ Department of Medicine, Laboratory of Molecular Biology, Cambridge University, Cambridge, United Kingdom.; ^3^ Mouse Informatics Group, Wellcome Trust Sanger Institute, Cambridge, United Kingdom.; ^4^ Zebrafish International Resource Center, Pathology and Health Services, Eugene, Oregon.

## Abstract

Managing the welfare of laboratory animals is critical to animal health, vital in the understanding of phenotypes created by treatment or genetic alteration and ensures compliance of regulations. Part of an animal welfare assessment is the requirement to record observations, ensuring all those responsible for the animals are aware of their health status and can act accordingly. Although the use of zebrafish in research continues to increase, guidelines for conducting welfare assessments and the reporting of observations are considered unclear compared to mammalian species. To support the movement of zebrafish between facilities, significant improvement would be achieved through the use of standardized terms to ensure clarity and consistency between facilities. Improving the clarity of terminology around welfare not only addresses our ethical obligation but also supports the research goals and provides a searchable description of the phenotypes. A Collaboration between the Wellcome Trust Sanger Institute and Cambridge University (Department of Medicine-Laboratory of Molecular Biology) has led to the creation of the zebrafish welfare terms from which standardization of terminology can be achieved.

## Introduction

The role of gene variation in human development, physiology, and disease is complex and our understanding is limited. The zebrafish is frequently the model system of choice to investigate the function of human genes.^1^ The zebrafish is a popular choice as a model because not only is it a vertebrate with a high degree of genetic similarity to humans but it has practical advantages that aid genetic studies. These practical advantages include the following: the ease by which they can be housed and cared for, their rapid development, ease of breeding, high fecundity, and their transparency allowing for rapid assessment of impact due to genetic alteration or drug treatment and ease of genetic change. The importance of genetically altered (GA) zebrafish to current biological research can be seen in genome wide projects to assess the role of every protein-coding zebrafish gene through a high-throughput mutagenesis and phenotyping project.^2^

As with all *in vivo* research, there is an ethical obligation to minimize the suffering of animals used, reduce the number of animals used where possible, and ensure as much information is gathered as possible from each animal used.^3^ To achieve this obligation, welfare assessments are conducted daily from which welfare concerns are recorded and the animal cared for accordingly. Welfare concerns can arise from environmental impact such as poor water quality or inadequate housing, including bacterial and parasitic infections.^4^ Genetic alteration or treatment can also have adverse effects including the creation of phenotypes such as loss of caudal fin, inability to feed due to jaw deformities, premature aging, and tumors. The description of such phenotypes should provide accurate information regarding the phenotypic characteristics and the welfare implications caused, allowing for humane end points to be established ensuring the animal does not suffer unnecessarily.^5^ These welfare concerns can be progressive and can have late onset or be initially sporadic. Standardized welfare assessments can support the differential between the two when assessing the following: welfare records over time, ensuring early signs of disease are seen, correct care provided at the earliest time point possible, ability to gain further knowledge of phenotypes, adherence to humane end points, ensure animals are checked and confirmed healthy to travel, provide information regarding the health of the animals being supplied, reduce the number of fish used, and provide the ability to retrospectively score severity.

Healthcare reports provide information regarding the health status of an animal and are essential to appropriately capture welfare concerns, however, when these concerns are not recorded using standardized terms, animal care information can be lost potentially leading to the animal experiencing unnecessary suffering, pain, and distress. To reduce inconsistencies of language used, facilities have created “local language” from which the animal carers and research staff define the terms to be used when recording health observations. While this ensures a standardized approach to describing an observation in-house it does not ensure consistency or interpretability between facilities. The consequences of such inconsistencies were first noted by the mouse community^6^ who highlighted that the use of nonstandardized terminology could lead to ethical welfare issues and impact on dissemination of research findings. With the rise of zebrafish transfers between facilities around the world, our community is also at risk of these consequences including inadequate quarantine procedures, inappropriate husbandry for specific strains, unexpected welfare concerns due to misinterpreted terms within the health report provided by the supplier, incomparable results between facilities and potential researchers not realizing they are researching the same phenotype.

Working in collaboration and with veterinarian advice, we have created a list of welfare terms to be used by animal care staff and researchers within a zebrafish facility. These terms create a standardized approach to ensure consistency in language used across facilities, and provide the capability to raise awareness of potential health concerns specific to genetic background or environmental impacts on the health of the animal. By standardizing the terms used, husbandry requirements can be shared as a community to ensure the welfare of the animal and create consistency across facilities.

## Welfare Assessment

The Code of Practice for the Housing and Care of Animals Bred, Supplied or Used for Scientific Purposes states that we must “have a strategy in place to ensure that the health status of the animals is maintained that safeguards animal welfare and meets scientific requirements.”^7^ To assist facilities in achieving this, welfare assessments are put in place, which consist of daily health checking of an animal, the recording of observations, and the appropriate action taken as required.

Recognizing signs of pain, suffering, and distress is essential to ensure action is taken at the earliest possible time point to alleviate the animal's suffering, ensure early signs of phenotypes are recorded, and to ensure that procedure end points are not exceeded. It is therefore necessary that animal care staff and researchers are trained and competent to perform this task, which includes the capability to recognize divergence from normal zebrafish appearance for that strain (e.g., bright color, flattened scales) and abnormal behavior (e.g., loss of balance or gasping at water surface). In the context of mice, Wells *et al.*^8^ proposed a structured assessment of the welfare of new GA lines to generate a “welfare profile,” which, once the line is established, would allow the monitoring to be focused on welfare indicators specific to that line.

Since 2001, a number of committees and organizations have published reports requesting that information on their creation, breeding, husbandry, and care should be collated for all GA animals.^5,9,10^ The Royal Society for the Prevention of Cruelty to Animals (RSPCA) initiated a GA Passport Working Group to develop a best practice booklet with guidance on the information required for all species^11^ to ensure that information remains with a genetically modified line through their life. The guidance is to include general information such as expected coat color and behavioral characteristics along with phenotypic abnormalities and observable traits with welfare implications. The Canadian Council on Animal Care has produced an example document that includes this information.^12^

By using the standardized zebrafish welfare terms, the GA passport can be used for the zebrafish community also, ensuring animals moved between facilities, regardless of species, are provided the care they require, “whoever cares for them throughout their lifetime.”

## Zebrafish Welfare Terms

**FIG. 1.**
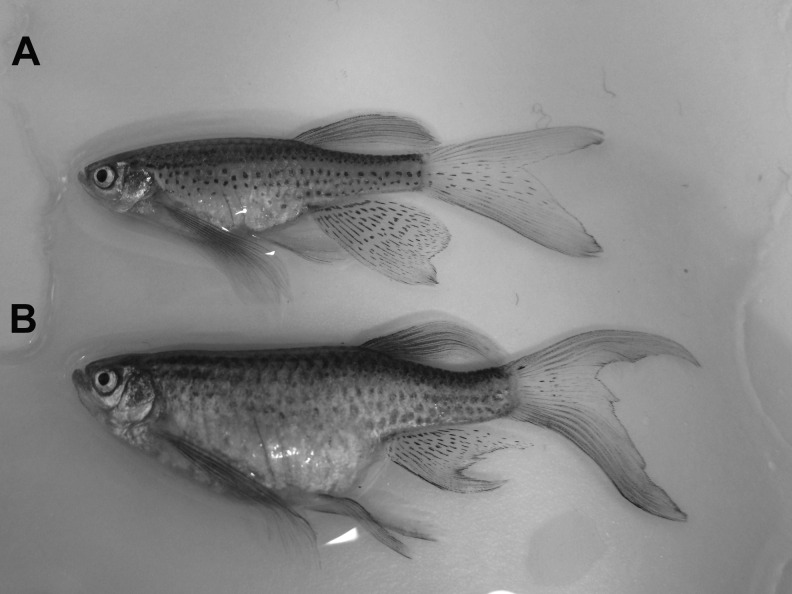
Example of a descriptive annotation of a zebrafish welfare concern. **(A)** Normal appearance of zebrafish, **(B)** female with enlarged abdomen, which would be annotated as *abdomen_general_distended.*

To ensure a standardized language is understood by all and searchable by bioinformatics, the welfare language for zebrafish needs to follow the following principles, which have been adapted from the article: Talking welfare: the importance of a common language^6^:

• Welfare assessments must consist of a description not a diagnosis. For example, if a female zebrafish has an enlarged abdomen, it must be described that way and not interpreted as being egg-bound (Fig. 1).
• The terminology used must be recognized by other facilities to ensure clarity. This includes considering international borders and specialities, for example, including veterinarians.
• Hierarchical descriptions that define the region, anatomical location, and observation will allow all facility staff and veterinarians to understand what has been observed in a standardized format. From this the impact and similar trends can be considered.
• The inclusion of meta-data, such as age, husbandry condition, and experimental procedures that the animal has undergone are necessary to provide a clear and full welfare assessment.

Following these principles, will allow in future a conversion of this standardized language to an ontology (where the language is standardized and the relationship between terms determined). Using these principles, a committee of experts including animal house managers, veterinarians, and animal care takers have developed a framework to organize the standardized language. The framework includes a hierarchal structure to give a description and location of the welfare issue to be captured. The language follows the structure of a typical welfare assessment, consisting of a general assessment followed by a nose to tail assessment. The first element of the language (“parameter”) captures whether the issue is general and associated with behavior or appearance or its gross location (e.g., Appearance, Behavior, Abdomen, Fin, etc.) (Table 1). This is followed by an optional “sub-parameter” that gives a more detailed location (e.g., for the parameter Abdomen you could have sub-parameters Anus, Scales/Skin, or General). The next element, captures the welfare indicators (e.g., obese or loss of scales). Again there is an option, when appropriate, for a welfare indicator subcategory (e.g., for a distended abdomen you could include soft or hard).

**Table 1.** Examples of the Standardized Welfare Terms

*Parameter*	*Sub parameter*	*Welfare indicators*	*Indicator sub category*	*Synonym*	*Definition*
Appearance	General	Loss of scales			Scales detached from body
Appearance	General	Lesion all over		Wound	
			Open-Abrasion		Damage to the skin consisting of loss of the epidermis and portions of the dermis but not the complete thickness of the skin.
			Open-Incision		A wound created by a sharp object. Edges are smooth and trauma to surrounding tissue is minimal.
			Open-Laceration		An irregular wound created by tearing of tissue. Damage to both superficial and underlying tissue is variable.
			Open-Puncture		A penetrating wound caused by pointed object.
			Closed-Contusion		Damage of the skin and/or underlying structures without breaking the skin for example, bruising, crush.
Appearance	General	Skin ulcers			Nonhealing erosions of skin.
Appearance	General	Multiple masses under skin		Swellings, raised areas, lumps	Abnormal appearance of masses of all descriptions (hard, soft, different shapes, etc.)
Appearance	General	Raised scales		Protruding scales	Scales protruding outward from body.
Appearance	General	Obese		Large, fat	Extremely fat, grossly overweight.
Appearance	General	Weight loss			Reduction in body weight compared to controls.
Appearance	General	Weight gain			Increase in body weight compared to controls.
Appearance	General	Thin		Emaciated, skinny	Lean or slender in form

In all languages, synonyms (a word or phrase that means exactly or nearly the same as another word or phrase in the same language) exist. As a result it is unsurprising that a common condition can be described using different terms. To increase the understanding across the community, we have included synonyms for each welfare term if available. We anticipate that this area of the language will evolve as the community provides further variations of a term.

**FIG. 2.**
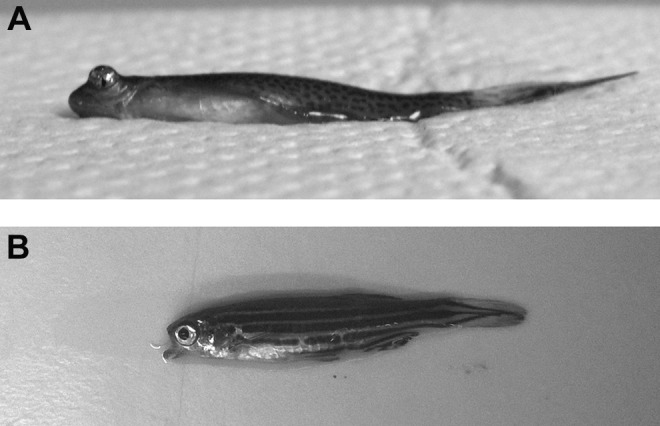
Examples of zebrafish welfare concern. **(A)** A zebrafish with an eye issue leading to the annotation *head_eyes_deformed.* **(B)** A zebrafish with a jaw issue leading to the annotation *head_mouth_malformed jaw*.

Alongside the language and synonyms, to provide additional clarity to aid consistent application, we have provided definitions and, when available, pictures of the terms they represent. For example, a zebrafish displaying an enlarged eye (Fig. 2A) would be assigned the annotation *head_eyes_deformed* while a fish displaying a malformed jaw (Fig. 2B) would be assigned *head_mouth_malformed jaw*. This document will be a living document hosted at the Zebrafish Model Organism Database Portal^13^ as a go to zebrafish resource (https://wiki.zfin.org/display/ZHWG/Zebrafish+Health+and+Welfare+Glossary+Home)^14^ where it will be updated periodically. As such, an e-mail contact is provided on the webpage to access a curator who can add images, synonyms, or new terms.

## Difficulties/Challenges

There is the potential to capture welfare to a more refined level. For example, there is the option to capture the location of a welfare issue when it is affecting one organ of which an animal has multiple (e.g., the left eye is enlarged). This fine level of information regarding specific location could be captured via the use of anatomic orientation terms (e.g., caudal or anterior). We have made the decision not to capture the information to this level of granularity after assessing the burden and the value returned. We felt this fine level detailed information would significantly increase the training needs for little improvement in welfare management.

An issue that arises is that at times the selection of the welfare term is subjective; for example—a fish presenting a swollen eye (head_eye_swollen) could be mistakenly defined by some as enlarged (head_eye_deformed_macro-opthalamia). Accumulation of observations will allow the definition of welfare issues to narrow. For this example, further observations will assist as an enlarged eye is a chronic and established condition where the fish is born with a larger eye, while a swollen eye is acute and intermittent and characterized by protruding from the eye socket. Furthermore, inconsistency arising from the subjective element can be minimized by careful training and the further refinement to the welfare terms to include pictures gathered from other facilities. This will assist with future training and ensure clarity of the correct terms to be used.

One of the challenges of selecting welfare terms is whether the term is localized or generalized. The choice depends on the extent of the condition. This, however, introduces another subjective element. Within the language we have assigned a general term (e.g., Appearance_General_lesions all over) for when the lesions appear in multiple places. This contrasts to situations where the lesion is localized, for example, Abdomen-Scales/Skin-lesion. Our advice is that if the animal care taker cannot clearly state the region then it becomes a global statement, for example, “general” or “group behavior.”

The GA passport intends to capture the welfare issues that arise specifically for that genetic alteration rather than incidental findings (expected welfare concerns for that background strain). An example would be curled operculums, which is seen commonly in zebrafish with a Tübingen background.^15^ While these incidental findings require management at a welfare level, the intention of the GA passport is to communicate the unexpected welfare issues.^6^ In the mouse community the expected welfare issues for a background strain issue are readily available from the Mouse Genome Informatics (MGI) website.^16^ For example, the C57BL/6 mouse strain is susceptible to obesity, which can be managed with a modified diet.^17^ However, to date this information was not available in the equivalent zebrafish resource and therefore could benefit from being communicated as a searchable referenced resource.

## Future Perspectives

This standardized language will need to evolve as our understanding of welfare progresses and we as a community recognize different impacts of genotype alteration or treatments. We also have the potential to consider associating these terms with potential solutions/support plans to share knowledge of how to manage these welfare needs. Though these terms are not a diagnosis, practical solutions/adaptations could be proposed.

As a standardized language, in the future, the terms could be associated with the zebrafish phenotyping ontology language^13^ where appropriate. By following the principles laid out in the section “Welfare Assessment” the potential will exist to convert this language to a fully defined ontology. Ideally, this would be a cross-species ontology as this would allow the use of existing knowledge and map relationships between observed phenotypes, which will lead to a better understanding of specific genetic phenotypes across species.

## Conclusions

The importance of welfare assessment is well established, however, the zebrafish research community embracing this shared standardized language will support the effective and efficient sharing of knowledge required to successfully rear and maintain stock, minimize detrimental welfare concerns, and reduce animal suffering. The standardized language as a training tool will help remove some of the subjectivity in interpretation of welfare issues. The drivers though are not just to meet our ethical obligations but that these welfare concerns indicate phenotyping knowledge that could support scientific discovery. With the use of a standardized language, the data has the potential to be accessible and searchable for future referencing and analysis. Adoption of standardized terms for describing and disseminating welfare concerns will better support animal care and scientific research.

## References

[1] Schier A Genomics: Zebrafish earns its stripes. Nature 2013;496:443–444 23594741 10.1038/nature12094

[2] Kettleborough R, *et al.* A systematic genome-wide analysis of zebrafish protein-coding gene function. Nature 2013;496:494–497 23594742 10.1038/nature11992 PMC3743023

[3] Johansen R, Needham J, Colquhoun D, Poppe T, Smith A Guidelines for health and welfare monitoring of fish used in research. Lab Anim 2006;40:323–340 17018205 10.1258/002367706778476451

[4] Kent M, Spitsbergen J, Matthews J, Fournie J, Murray K, Westerfield M: ZIRC health services zebrafish disease manual, 2012 http://zebrafish.org/health/diseaseManual.php (accessed 1218, 2015)

[5] Rose M, Everitt J, Hedrich H, Schofield J, Dennis M, Scott E, *et al.* ICLAS Working Group on Harmonization: International guidance concerning the production care and use of genetically-altered animals. Lab Anim 2013;47:146–152 23563121 10.1177/0023677213479338

[6] Bussell J, Wells S Talking welfare: The importance of a common language. Mamm Genome 2015;26:482–485 26286857 10.1007/s00335-015-9591-x PMC4602052

[7] UK Government: Code of practice for the housing and care of animals bred, supplied, 2014 www.gov.uk/government/uploads/system/uploads/attachment_data/file/388535/CoPanimalsWeb.pdf (accessed 1213, 2015)

[8] Wells D, Playle L, Enser W, Flecknell P, Gardiner M, Holland J, *et al.* Assessing the welfare of genetically altered mice. Lab Anim 2006;40:111–114 16600070 10.1258/002367706776318971

[9] Hawkins P, Morton D, Burman O, Dennison N, Honess P, Jennings M, *et al.* A guide to defining and implementing protocols for the welfare assessment of laboratory animals: Eleventh report of the BVAAWF/FRAME/RSPCA/UFAW Joint Working Group on Refinement. Lab Anim 2011;45:1–13 21123303 10.1258/la.2010.010031

[10] UK Government: Report of the animal procedures committee for 2007, 2008 www.gov.uk/government/publications/report-of-the-animal-procedures-committee-for-2007 (accessed 1213, 2015)

[11] RSPCA GA Passport Working Group: GA passports the key to consistent animal care. Research Animals Department RSPCA, West Sussex, 2010 www.science.rspca.org.uk/sciencegroup/researchanimals/implementing3rs/gapassport (accessed 1217, 2015)

[12] CCAC Three Rs Microsite: Animal passports, 2014 http://3rs.ccac.ca/documents/en/Animal_Passports (accessed 128, 2015)

[13] Sprague J, *et al.* The Zebrafish Information Network (ZFIN): The zebrafish model organism database. Nucleic Acids Res 2003;31:241–243 12519991 10.1093/nar/gkg027 PMC165474

[14] Wiki.zfin.org: Zebrafish health and welfare glossary – General information – ZFIN Community Wiki, 2015 https://wiki.zfin.org/display/ZHWG/Zebrafish+Health+and+Welfare+Glossary+Home (accessed 1221, 2015)

[15] Zebrafish.org: ZIRC frequently asked questions, n.d. https://zebrafish.org/documents/faq.php#15 (accessed 17, 2016)

[16] Eppig JT, Blake JA, Bult CJ, Kadin JA, Richardson JE; The Mouse Genome Database Group. The Mouse Genome Database (MGD): Facilitating mouse as a model for human biology and disease. Nucleic Acids Res 2015;43(Database issue):D726–D736 25348401 10.1093/nar/gku967 PMC4384027

[17] Surwit RS, Feinglos MN, Rodin J, Sutherland A, Petro AE, Opara EC, *et al.* Differential effects of fat and sucrose on the development of obesity and diabetes in C57BL/6J and A/J mice. Metabolism 1995;44:645–651 7752914 10.1016/0026-0495(95)90123-x

